# Immunoexpression of placental growth factor (PlGF) and soluble FMS-like tyrosine kinase 1 (sFlt-1) in the placental bed of preeclamptic women of African ancestry living with HIV infection

**DOI:** 10.1007/s00418-024-02341-6

**Published:** 2024-11-23

**Authors:** Zinhle P. Mlambo, Motshedisi Sebitloane, Thajasvarie Naicker

**Affiliations:** 1 Optics and Imaging Centre, Nelson R. Mandela School of Medicine, Doris Duke Medical Research Institute, College of Health Sciences, University of KwaZulu-Natal, Durban, South Africa; 2 Department of Obstetrics and Gynaecology, Nelson R. Mandela School of Medicine, University of KwaZulu-Natal, Durban, South Africa

**Keywords:** Preeclampsia, PlGF, sFlt-1, HIV, Immunohistochemistry

## Abstract

Preeclampsia, a severe pregnancy complication linked to defective placentation, poses significant maternal risks and is characterized by dysregulated angiogenic factors, including placental growth factor (PlGF) and soluble fms-like tyrosine kinase-1 (sFlt-1). Women with HIV/AIDS and receiving ART may face an increased susceptibility to preeclampsia development due to immunological and angiogenic imbalance. This study investigates the immunoexpression of these factors in the context of HIV-associated preeclampsia, utilizing morphometric image analysis. The study cohort comprised 180 women, including 60 normotensive and 120 preeclamptic participants, further stratified by HIV status and gestational age (early-onset PE [EOPE] < 34 weeks and late-onset PE [LOPE] ≥ 34 weeks). Placental bed tissues were immunostained with mouse anti-human sFlt-1 and PlGF antibodies, and the results were analyzed using Zeiss Axio-Vision and GraphPad Prism software. sFlt-1 levels showed no significant overall difference between preeclamptic and normotensive women (*p* = 0.8661), though slightly increased in the preeclamptic myometrium, independent of HIV status. However, sFlt-1 levels were significantly higher in EOPE compared to both normotensive and LOPE groups. PlGF immunostaining also showed no significant overall difference (*p* = 0.7387) but was notably lower in preeclamptic pregnancies and significantly higher in EOPE compared to LOPE. HIV status did not significantly impact sFlt-1 or PlGF levels, although sFlt-1 was slightly higher in HIV-negative women, while PlGF was marginally higher in HIV-positive women. These findings highlight the complex role of angiogenic factors in preeclampsia pathophysiology and suggest that antiretroviral therapies (ARTs) may contribute to the dysregulation of these factors due to a heightened immune milieu.

## Introduction

Preeclampsia (PE) is a complex hypertensive disorder of pregnancy, affecting approximately 5–8% of pregnancies worldwide (Khaliq et al. 2023; Ni and Shkurat 2022). Worldwide, it is a leading cause of maternal and perinatal morbidity and mortality accounting for approximately 14% of maternal deaths and contributing for adverse outcomes such as preterm births, low birth weight, placental abruption, and even maternal organ dysfunction (Chang et al. 2023). Preeclampsia is believed to arise from the interaction among genetic factors, impaired placentation, and maternal immune maladaptation (Dimitriadis et al. 2023; Lian et al. 2022). The abnormal placentation emanates from deficient trophoblast migration and a lack of spiral artery remodeling within the myometrium (Onyangunga et al. 2018). Moreover, it is widely accepted that angiogenic factors play a key role in the process of trophoblast invasion and the physiological transformation of spiral arteries during normal human pregnancy (Lecarpentier and Tsatsaris 2016).

The defective placentation of preeclampsia predisposes patients to placental ischemia and elevated oxidative stress (Jena et al. 2020; Staff et al. 2022). These processes disrupt the delicate balance of angiogenesis, inflammation, and endothelial function, ultimately leading to the clinical manifestation of preeclampsia (Jena et al. 2020). Previous studies suggest that the occurrence of preeclampsia may be affected by immunosuppressive conditions such as HIV/AIDS (Kalumba et al. 2013; Landi et al. 2014).

Women living with HIV are believed to have an increased risk of developing preeclampsia compared to HIV-naive women (Bhorat et a*.* 2023; Kourtis et al. 2007). The mechanisms underlying this association are not completely understood, but it is believed to be multifactorial, involving immunological, inflammatory, and angiogenic dysfunction (Naicker et al. 2019). This hypoxic microenvironment creates an imbalance of circulating angiogenic [vascular endothelial growth factor (VEGF), placental growth factor (PIGF)] and antiangiogenic factors [soluble fms-like tyrosine kinase-1 (sFlt-1), soluble endoglin (sEng)] (Qi . 2022). More specifically, decreased levels of PlGF and increased levels of sFlt-1 prevail in preeclampsia (Padayachee et al. 2019; Rana . 2022; Sa et al. 2020).

Placental growth factor and sFlt-1 are key angiogenic factors that play a crucial role in normal placental development (Stalmans . 2003; Zeisler et al. 2016) by promoting angiogenesis and vasculogenesis, thereby influencing endothelial cell proliferation, migration, and survival (Rautiainen et al. 2021; Stalmans . 2003). In contrast, sFlt-1 functions as a potent anti-angiogenic molecule that acts as a decoy receptor for VEGF and PlGF, preventing their binding to specific receptors (Carrasco-Wong et al. 2021).

The placental bed is a key site for maternal-fetal communication and contains various cell types, including trophoblasts and endothelial cells, which are involved in the regulation of angiogenesis and placentation during gestation (Bulmer et al. 2010; Veerbeek et al. 2015). Most studies have focussed on the expression of angiogenic factors in the placenta, but their expression and potential role in the placental bed require investigation (Kim et al. 2012; Kim et al. 2012). Notably, the immunoexpression and localization of PlGF and sFlt-1 within the placental bed tissue may provide valuable insights into the underlying pathophysiology of pre-eclampsia associated with HIV infection. Morphometric image analysis is a powerful tool that allows for quantitative assessment of protein expression and localization within tissues by analyzing the spatial distribution and intensity of immunostaining of antigens compared to conventional subjective interpretations (Lattouf et al. 2014; Rojo et al. 2009). Moreover, it enables the extraction of quantitative data, which may be further correlated with clinical characteristics or outcomes. This study aims to investigate the immunoexpression of angiogenic factors (PlGF and sFlt-1) in the placental bed of HIV-associated preeclampsia using morphometric image analysis.

## Materials and methods

### Study population

This study received institutional approval (BE 040/12, BCA 338/17), hospital manager’s consent, and informed consent from all participants. The placental bed was obtained from *n* = 180 pregnant women immediately after delivery at a large regional hospital (Prince Mshiyeni Hospital in Umlazi, KwaZulu-Natal, South Africa). The study population consisted of normotensive pregnant (*N*, *n* = 60) and preeclamptic (*n* = 120) women. The latter group was then stratified by gestational age into early-onset PE (< 34 weeks of gestation, *n* = 60) and late-onset PE (LOPE, > 34 weeks of gestation, *n* = 60). Additionally, each of these groups was further sub-stratified by HIV status into HIV-negative normotensive pregnant (N – ve, *n* = 30), HIV-positive normotensive pregnant (N + ve, *n* = 30), HIV-negative early onset PE (EOPE – ve, *n* = 30), HIV-positive early onset PE (EOPE + ve, *n* = 30), HIV-negative late-onset PE (LOPE – ve, *n* = 30), and HIV-positive late-onset PE (LOPE + ve, *n* = 30) groups.

Pregnant women > 18 years old were recruited according to specific inclusion and exclusion criteria during their antenatal period at a public health facility in South Africa. Preeclampsia was defined by the presence of new-onset hypertension (systolic blood pressure ≥ 140 mmHg and/or a diastolic blood pressure ≥ 90 mmHg) measured on two occasions a few hours apart and proteinuria (protein ≥  + 1 on urinary dipstick method with/without a 24-h urine protein quantitative test of at least 300 mg/dl) occurring at or after the 20th week of gestation (Magee et al. 2022). Early-onset preeclampsia occurs at < 34 weeks gestation, whereas late-onset preeclampsia develops at or after 34 weeks of gestation (Dimitriadis et al. 2023; Teka et al. 2023). All study participants were delivered by elective cesarean section. Clinical and demographic data were recorded on a structured data form. Exclusion criteria included chronic hypertension, intrauterine death, chorioamnionitis, diabetes, autoimmune diseases, history of seizures, and cardiovascular disease.

### Immunohistochemistry

*Sample collection*: In the primary study, a segment from the central region of each placental bed was excised, taking care to avoid areas with macroscopic infarction. The samples were immediately fixed in a 10% buffered formaldehyde solution for 30 min.

*Fixation, processing, and microtomy*: Placental tissue was dehydrated using an ascending ethanol series (70%,95%, and 100%), cleared with xylene, and infiltrated with paraffin wax using an automated tissue processor (Sakura 5, Torrance, CA, USA). The tissue was then embedded in an embedding station (Leica EG 1160 embedding station, Germany) and archived until the immunostaining procedure. Blocks were trimmed, and 3–4 µm sections were cut on a rotatory microtome (Leica RM2135, UK). Sections were floated on a 50 °C water bath (Leica HI1210, Leica Biosystems, UK), collected onto X-tra adhesive coated slides (Leica Biosystems, UK), and heat-fixed on a 60 °C hot plate (Sakura, USA) (15 min). This was followed by deparaffinization and rehydration in a descending series of ethanol.

*Immunohistochemistry*: To immunostain PlGF and sFlt-2 within the placental bed, the PlGF antibody and VEGF receptor 1 primary antibody (Themofisher) were used along with Dako EnVision FLEX mini kit (K8023; Dako, Denmark). The sections were de-paraffinized, hydrated, and rinsed in tap water before incubation in a pre-heated low pH 6 target retrieval solution for 7 min. The slides were then cooled at room temperature (20 min), rinsed, and placed in a wash buffer solution (DM831, Dako, Denmark) (5 min). Thereafter, the sections were circled with a delimiting hydrophobic pen (Dako, Denmark). To block endogenous peroxidase activity, a peroxidase-blocking reagent (SM801, Dako, Denmark) in a humidity chamber was used (30 min). Thereafter, the samples were washed with "wash buffer" for 5 min and incubated with the primary antibody (PlGF,1/1000 and sFlt-1,1:200 dilution, 30 min). The sections were washed and incubated with horse radish peroxidase conjugate (HRP) conjugated secondary antibody (20 min) followed by rinsing in wash buffer. Antibody expression was detected by incubation in 3, 3’ diaminobenzidine (DAB) chromogen. The slides were rinsed, immersed in Mayer’s hematoxylin (5 min each), and rinsed in a wash buffer. Thereafter, the samples were dehydrated, cleared in xylene, and the cover-slipped. The placenta served as the positive control. Replacement of the primary antibody with a buffer and non-immune serum of the same IgG class as the primary served as the negative control.

*Morphometric analysis:* The Axioscope A1 microscope (Carl Zeiss, Germany) was used to view the prepared placental tissue sample, and a random selection of four fields of view per slide was analyzed at an objective magnification of 20. AxioVision Image Analysis Software version 4.8.3 (Carl Zeiss, Germany) was used to acquire, capture, process, and analyze the images. ImageJ was used as a quantitative measuring tool, and PlGF and sFlt-1 immunoexpression were evaluated as a percentage of immunostaining per frame area.

*Data analysis techniques:* An Excel database was created to compare PlGF and sFlt-1 expression between pregnancy types, by HIV status and across all groups,

*Statistical analysis:* Sample size was determined using the Cohens' formula. Results were tested for normality before analysis. Two-way ANOVA was performed to examine the effect of HIV status (HIV – vs. HIV +) and pregnancy type (normotensive pregnant vs. pre-eclamptic) across all groups. Bonferroni post hoc test was used to further assess the data between subgroups and sub-categories. A probability level of *p* < 0.05 was considered statistically significant. All statistical analyses were conducted using GraphPad PrismTM version 5.01 (San Diego, CA, USA).

## Results

### Morphometric image analysis of PlGF and sFlt-1 immunoexpression within placental bed tissue:

**Fig. 1:**
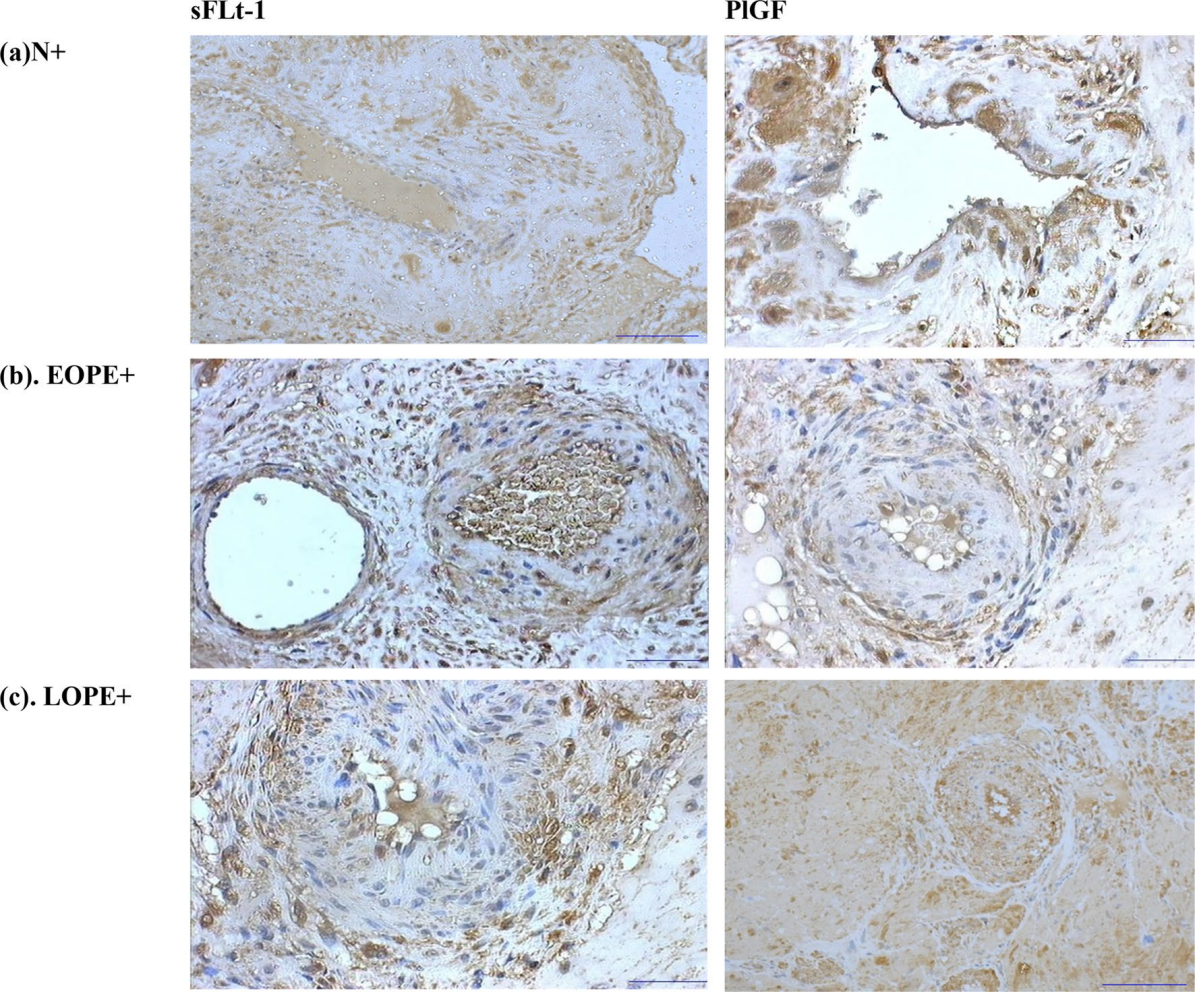
Immunohistochemical localization of sFlt-1 and PlGF protein in the human placental bed of a preeclamptic woman in the **a** normotensive HIV positive (N+), **b** early onset preeclampsia HIV positive (EOPE+), and **c** late-onset preeclampsia HIV positive (LOPE+). Magnification: 100 µm.

Migratory invasive interstitial trophoblast cells were quantified within the decidua of both the normotensive and preeclamptic groups. The decidua appeared fragmented in most biopsies. Decidual cells appeared as large ovoid cells. Several different cell types in both PE and normotensive control groups were immunostained for PlGF and sFlt-1 including extravillous trophoblast cells, migratory trophoblast cells, giant cells, and endothelial cells. Trophoblast cells were immunostained for both PlGF and sFlt-1, albeit lower in the aforementioned antibody. Trophoblast giant cells appeared as multinucleated cells and varied in their immunoreactive pattern (Fig. 1). Spiral arteries displayed total physiological conversion in all normotensive groups where their muscular wall was replaced by a fibrinoid-like material.

### Morphometric analysis of sFlt-1 and PlGF immunostaining

The study successfully obtained true placenta bed biopsies across all subgroups, including N + ve, N − ve, EOPE − ve, EOPE + ve, LOPE − ve, and LOPE + ve. In all groups, sFlt-1 and PlGF were consistently localized to the endothelial cells of both the arterial supply and venous drainage within the conducting villi, irrespective of HIV status. However, the level of immunoreactivity varied, with preeclamptic groups showing stronger staining than normotensive, regardless of HIV infection. Interestingly, sFlt-1 and PlGF immunostaining were low to absent in trophoblast cell populations, such as syncytiotrophoblast and cytotrophoblast cells. This indicates that these angiogenic markers mainly target endothelial cells in the placental bed rather than trophoblastic cells. Additionally, numerous macrophage-like cells stained for sFlt-1 and PlGF were observed in the veins, arteries, and capillaries of the fetal circulation as well as in the intervillous space of the maternal circulation. This distribution was significantly lower in the normotensive HIV-negative group compared to the other groups.

**Fig. 2:**
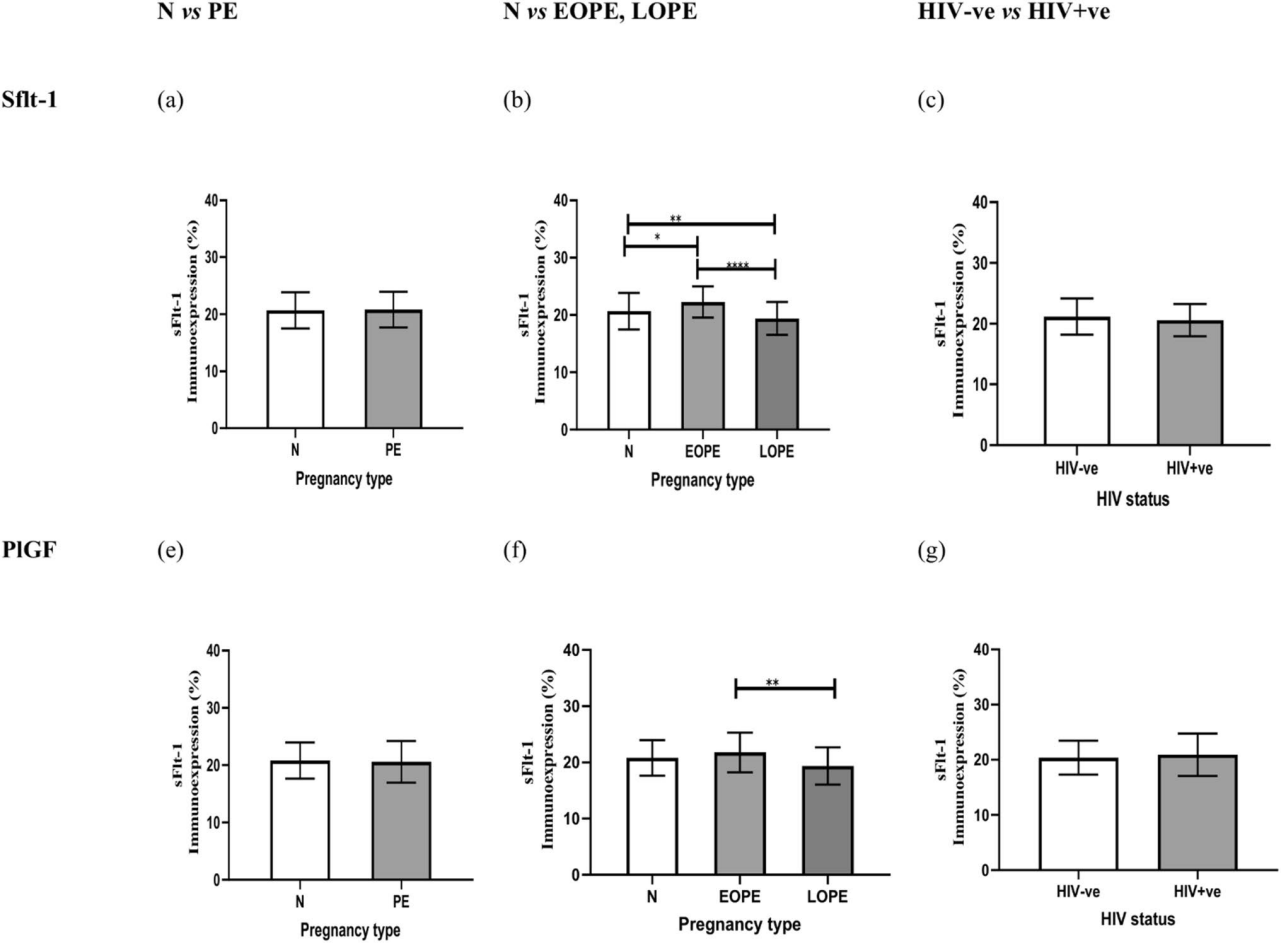
Graphical representation of PlGF and sFlt-1 immunoexpression comparisons between Pregnancy types, HIV status, and across all groups within the spiral arteries of the myometrium. N normotensive, PE Preeclampsia, EOPE early onset preeclampsia, LOPE late-onset preeclampsia, HIV-ve Human immunodeficiency virus-negative, HIV + ve Human immunodeficiency virus-positive, N − ve normotensive HIV negative, N + ve normotensive HIV positive, EOPE − ve Early-onset pre-eclampsia HIV negative, EOPE + ve- Early onset preeclampsia HIV positive, LOPE-ve Late-onset preeclampsia HIV negative, LOPE + ve Late-onset preeclampsia HIV positive. Asterisks (*) denote significance: *p < 0.05, **p < 0.01, ***p < 0.001, and ****p < 0.0001

There was no significant difference in the immunoreactive of sFlt-1, in preeclamptic compared to normotensive pregnant women (*p* value = 0.8661), however, there was a slight elevation within the myometrium of preeclamptic (20.83 ± 3.134) compared to normotensive (20.67 ± 3.188) women, irrespective of HIV status. When stratified according to gestational age (EOPE and LOPE) compared to normotensive pregnant women, a significant difference was noted *p*< 0.0001****using the Kruskal–Wallis test across the three groups. When further analyzed using Dunn's multiple comparisons test according to gestational age, the immunostaining of sFlt-1 was noted to be significantly decreased in normotensive pregnant women (20.67 ± 3.188) compared to EOPE subgroup (≤ 34 weeks gestation) (22.27 ± 2.707), p = 0.0140* and significantly increased compared to the LOPE subgroup (> 34 weeks gestation) [(19.39 ± 2.880),  *p*.0054**]. when comparing the two sub-stratified preeclamptic groups irrespective of their HIV status there was a significant increase difference in EOPE (22.27 ± 2.707) compared to LOPE (19.39 ± 2.880), *p*alue < 0.0001**** (Fig. 2).

The field area percentage of PlGF immunostaining within the myometrium of a placental bed of normotensive versus preeclamptic women was not significantly different; nevertheless, there was a decrease in the preeclamptic (20.58 ± 3.624) compared to normotensive (20.82 ± 3.165), *p* = 0.7387 women, irrespective of HIV status. When stratified according to gestational age (EOPE and LOPE) compared to normotensive pregnant women, a significant difference was noted (*p* = 0.0019**) using the Kruskal-Wallis test across the three groups. When further analyzed using Dunn's multiple comparisons tests, PlGF immunoreactivity showed no significant difference in normotensive (20.82 ± 3.165) compared to EOPE (21.79 ± 3.54), *p* = 0.2832; however, there was a slight upregulation of PlGF in the EOPE group with a decline in the LOPE (19.37 ± 3.312) group, *p* = 0.0806. When EOPE (21.79 ± 3.540) is compared to LOPE (19.37 ± 3.312), PlGF immunoexpression was significantly different (*p* = 0.0013**).

**Fig. 3:**
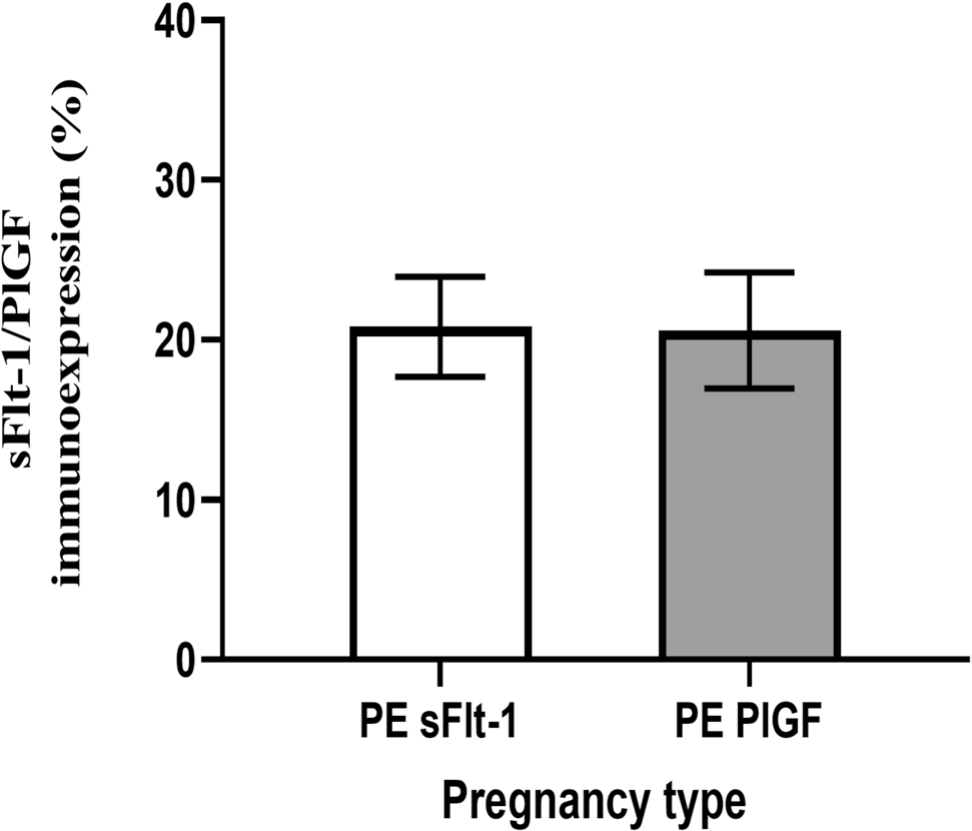
Graphical representation of ratio of sFlt-1 to PlGF immunoexpression in preeclamptic group

*HIV status irrespective of pregnancy type*: Based on HIV status, sFlt-1 immunoreactivity was similar across the study population (*n* = 180) irrespective of pregnancy type; however, it was higher in HIV – ve (21.17 ± 2.982) compared to HIV + ve (20.60 ± 2.671), *p* = 0.1411, groups. Similarly, PlGF immunostaining was not significantly different in HIV – ve (20.40 ± 3.072) compared to the HIV + ve (20.93 ± 3.827), *p* = 0.3042, irrespective of pregnancy type (Fig. 3).

### Across all groups

There was a significant difference across all groups *p* < 0.0001. Dunn's multiple comparisons tests showed significant downregulation of sFlt-1 immunoreactivity in N + ve (20.84 ± 2.204) compared to EOPE – ve (22.85 ± 2.540) groups, *p* = 0.0487^*^; however, EOPE – ve (22.85 ± 2.540) immunostaining compared to LOPE – ve (19.52 ± 3.265) was significantly upregulated, *p* < 0.0001^****^. sFlt-1 immunostaining was significantly different between EOPE + ve (21.68 ± 2.782) compared to LOPE – ve (19.52 ± 3.265) groups,* p* = 0.0211; furthermore, comparing EOPE + ve (21.68 ± 2.782) to LOPE + ve (19.26 ± 2.487) showed a significant difference *p* = 0.0045^**^ between the two groups.

PlGF immunostaining showed a significant difference between EOPE + ve (22.85 ± 3.786) compared to LOPE + ve (19.41 ± 3.510), *p* = 0.0056^**^. A minimal significant increase of PlGF in EOPE + ve (22.85 ± 3.786) compared with LOPE – ve (19.32 ± 3.160), *p* = 0.0029^**^ was noted. Dunn's multiple comparison tests showed that PlGF immunoreactivity was decreased in N – ve (21.13 ± 2.874) compared with EOPE + ve (22.85 ± 3.786), *p* = 0.9683, and increased compared to LOPE – ve (19.3 ± 3.160), *p* = 0.9107. A downregulation of PlGF was noted when comparing N + ve (20.52 ± 3.455) and EOPE + ve (22.85 ± 3.786), *p* = 0.1694. No significant difference in PlGF was noted in EOPE – ve (20.74 ± 2.977) compared to EOPE + ve (22.85 ± 3.786),* p* = 0.3192). However, there was a slight increase in immunoexpression of PlGF in EOPE + ve placental beds.

*sFlt-1 compared to PlGF immunoreactivity in the preeclamptic group*: The was no significant difference between sFlt-1(20.83 ± 3.134) and PlGF (20.58 ± 3.624), *p* = 0.8854; however, there was a slight increase in sFlt-1–1 immunoexpression compared to PlGF (Fig. 3).

### Clinical characteristics

Patient demographics of the study population at term are shown in Table 1 as the median and interquartile range (IQR) because of their non-parametric distribution. Maternal age (*p* < 0.0001), maternal weight (*p* = 0.0221), systolic pressure (*p* < 0.0001), diastolic pressure (*p* < 0.0001), gestational weight (*p* < 0.0001), and gestational age (*p* < 0.0001) were significantly different across the study groups. There was no significant difference in gravidity and parity, respectively (Table 1).

**Table 1:** Patient demographics and clinical data across all groups [N − ve, N + ve, EOPE − ve, EOPE + ve, LOPE − ve, and LOPE + ve]

Patient data	N – ve (*n* = 60)	N + ve (*n* = 60)	EOPE – ve (*n* = 60)	EOPE + ve (*n* = 60)	LOPE – ve (*n* = 60)	Lope + ve (*n* = 60)	*p*-value	*P *value (across all groups)
Gestational age (weeks)								
N − ve vs. EOPE − ve N − ve vs. EOPE + ve N − ve vs. LOPE − ve N − ve vs. LOPE + ve N + ve vs. EOPE − ve N + ve vs. EOPE + ve N + ve vs. LOPE − ve N + ve vs. LOPE + ve	38.00 (37.00–39.00)	37.00 (36.00–39.00)	30.00 (25.75–34.50)	33.00 (30.00–35.00)	35.50 (34.00–37.00)	35.00 (33.00–36.00)	< 0.0001**** 0.0099** < 0.0001**** < 0.0001**** < 0.0001****	< 0.0001****
Systolic BP (mmHg)								
N − ve vs. EOPE − ve	111.0 (106.0–116.8)	111.0 (105.0–117.0)	151.0 (143.0–160.0)	148.0 (136.0–161.0)	154.0 (146.5–173.0)	145.0 (141.8–153.3)	****	< 0.0001****
N − ve vs. EOPE + ve							****	
N − ve vs. LOPE – ve							****	
N − ve vs. LOPE + ve							****	
N + ve vs. EOPE − ve							****	
N + ve vs. EOPE + ve							****	
N + ve vs. LOPE − ve							****	
N + ve vs. LOPE + v							****	
Diastolic BP (mmHg)								
N − ve vs. EOPE − ve	71.00 (64.50–79.00)	70.00 (64.00–76.00)	95.50 (91.00–104.3)	92.00 (90.00–98.00)	106.0) 101.0 (94.00–108.0)	96.00 (91.00–98.25)	****	< 0.0001****
N − ve vs. EOPE + ve							****	
N − ve vs. LOPE − ve							****	
N − ve vs. LOPE + ve							***	
N + ve vs. EOPE − ve							****	
N + ve vs. EOPE + ve							*	
N + ve vs. LOPE − ve							**	
N + ve vs. LOPE + ve							****	
Maternal weight (kg)								
LOPE − ve vs. LOPE + ve*	73.70 (63.25–97.77)	67.30 (55.25–85.38)	67.20 (60.25–91.00)	81.00 (68.48–87.90)	66.00 (55.75–81.00)	85.35 (69.90–95.25)	*	0.2221*
Maternal age (years)								
N + ve vs. LOPE − ve	25 (22–30)	28 (25.00 – 31.00)	23 (20–27)	32 (25–37)	23 (19–26)	28 (25–34)	****	< 0.0001****
EOPE − ve vs. EOPE + ve							*	
EOPE − ve vs. LOPE + ve							**	
EOPE + ve vs. LOPE – ve							*	
LOPE − ve vs. LOPE + ve							***	
Gestational weight (kg)								
N − ve vs. EOPE − ve	3.150 (2.975–3.585) 2.900	3.100 (2.600–3.550)	2.100 (1.625–2.538)	1.900 (1.300–2.500)	3.000 (2.723–3.213)	(2.300–3.120) < 0.0001	****	< 0.0001****
N − ve vs. EOPE + ve							****	
N + ve vs. EOPE − ve							****	
N + ve vs. EOPE + ve							****	
EOPE − ve vs. LOPE − ve							****	
EOPE − ve vs. LOPE + ve							*	
EOPE + ve vs. LOPE − ve							**	

Across the population group (*n* = 180), the Kruskal-Wallis (K-W) test detected significance for maternal age (*p* < 0.0001), weight (*p* = 0.0221), systolic and diastolic pressure (*p* < 0.0001), and gestational weight and age (*p* < 0.0001). The Dunn's multiple comparison test further calculated the significance between the PE groups stratified by HIV, indicated in Table 1

## Discussion

This study reports no significant difference but a slightly elevation of sFlt-1 immunostaining within the placental bed of preeclamptic compared to normotensive pregnancies. This is an expected result as it is widely accepted that preeclampsia is associated with defective placentation (Fillion et al*,* 2021; Garrido-Gomez et al. 2017; Gui et al. 2020). More specifically, in PE the luminal diameter of spiral arteries remains of small caliber (Nobis et al. 2020; Sankar et al. 2013); hence, the field area percentage of endothelial cells is lower. Moreover, trophoblast cells are absent within these non-physiologically converted spiral arteries in PE. We also report a significant decline of sFlt-1 immunostaining within spiral arteries of EOPE and LOPE (*p* < 0.0001) compared to the normotensive pregnant group. Of the two subtypes of PE (EOPE and LOPE), EOPE is associated with deficient trophoblast invasion together with the total absence of myometrial spiral artery remodeling (Pillay and Naicker 2020).

This study also reports no significant difference with a decrease of PlGF immunostaining within the placental bed of PE compared to normotensive pregnancies. Compared to sFlt-1, the intensity of immunoreactivity of PlGF was reduced across the study population. Of note, PlGF is a pro-angiogenic molecule. Therefore, in the large flaccid remodeled spiral arteries of normotensive pregnancies, it is expected to have a larger surface area of endothelial cells compared to the non-physiological converted spiral arteries in the myometrium of PE. Previous literature has also demonstrated a reduction of PlGF in favor of sFlt-1 in PE compared to normotensive pregnancies, albeit within the placenta (Govender et al. 2013). Moreover, the absence of intramural trophoblast cells in PE supports our observed reduction of PlGF immunoreactivity. A notable decrease in PlGF immunostaining was noted in spiral arteries within the myometrium of normotensive pregnancies compared to EOPE (*p* = 0.4248) and an increase in LOPE (*p* = 0.1209) pregnancies. Among the two subtypes of preeclampsia, EOPE serves as a paradigmatic manifestation of preeclampsia, highlighting the pivotal role of dysregulated angiogenesis in its pathophysiology (Cele et al. 2018). The notable increase in PlGF expression observed in EOPE compared to the normotensive group in our study may be linked to the abnormal morphology of the placental bed resulting from altered blood flow dynamics.

To our knowledge, this is the first study to report on the immunostaining of PlGF and sFlt-1 within the placental bed of pregnancies complicated by PE comorbid with HIV infection in women of African ancestry. sFlt-1 has been extensively investigated as a key protein that may be involved in the etiology or as a secondary phenomenon of PE within the placenta only since studying the placental bed requires invasive procedures (Maynard et al. 2003; Mcelwain et al. 2020).

Another main finding of the study was the non-significant elevation of sFlt-1 within the myometrium of the placental bed in the HIV + ve compared to HIV – ve (*p* = 0.1411), irrespective of pregnancy type. It is a standard-of-care procedure in SA for all HIV-infected women to receive ART and prophylaxis to prevent mother-to-child HIV transmission (PMTCT) (Akinsanya et al. 2017; Wessels et al. 2020). In contrast to sFlt-1, we report a similar immunoreactivity of PlGF between the HIV-positive vs. HIV-negative (*p* = 0.3042) group. Of note, ART serves to restore the immune response, ameliorating the potent pro-angiogenic effect of HIV (Isaguliants et al. 2021). HIV is a powerful pro-angiogenic factor due to the homology of a shared lys-ala residue of its accessory protein Tat with the VEGF protein (Spector et al 2019). Moreover, the HIV-1 accessory and matrix proteins are proponents of the dysregulation of hypoxia, apoptosis, oxidative stress, angiogenesis, and cell invasion (Mazzuca et al. 2018; Singh et al. 2023). To date, there is no available literature on the subcellular localization of sFlt-1 and PlGF within the placental bed of HIV-positive patients. The underlying mechanism behind the observed outcome requires greater clarity. Nonetheless, it is hypothesized that HIV infection may have a protective effect through mechanisms such as immune suppression or the strong resemblance of the HIV tat protein to VEGF (Singh et al. 2023). Alternatively, the neutralization may be insufficient within the heightened immune milieu of PE.

Across all groups, sFlt-1 levels were significantly downregulated in normotensive HIV-negative women compared to EOPE HIV-negative women, suggesting an upregulation of anti-angiogenic factors in EOPE. Conversely, EOPE HIV-negative women exhibited higher sFlt-1 levels than LOPE HIV-negative and LOPE HIV-positive women, indicating that sFlt-1 is more prominently involved in EOPE rather than LOPE, irrespective of HIV status. Notably, EOPE HIV-positive women showed higher sFlt-1 levels compared to LOPE HIV-negative and LOPE HIV-positive women, reinforcing the association of sFlt-1 upregulation with EOPE. Similarly, a significant increase in the immunoexpression PlGF was noted in EOPE HIV-positive women compared to LOPE HIV-positive and LOPE HIV-negative groups. Normotensive HIV-negative women exhibited lower PlGF levels compared to EOPE HIV-positive women, indicating that preeclampsia and HIV together may exacerbate angiogenic imbalance (Padayachee et al., 2019). HIV infection is known to affect angiogenic and downstream cell transduction pathways (Mtshali et al. 2020; Padayachee et al. 2019). HIV proteins, such as gp120, can induce endothelial cell apoptosis and dysregulate angiogenic factors, contributing to vascular complications (Mazzuca et al. 2018; Seeherman and Suzuki 2021). HIV-related chronic inflammation and immune activation may further disrupt the angiogenic balance, potentially exacerbating preeclampsia symptoms. The interplay between HIV and angiogenic factors like sFlt-1 and PlGF in the placental bed highlights a complex mechanism where both viral infection and preeclampsia can lead to significant endothelial and placental dysfunction.

Despite no statistically significant difference noted between sFlt-1 and PlGF levels (*p* = 0.8854), there is a slight increase in sFlt-1 compared to PlGF immunoexpression. Previous studies have highlighted the importance of the sFlt-1/PlGF ratio as a biomarker for diagnosing and predicting the severity of preeclampsia (Zeisler et al. 2016). An elevated sFlt-1/PlGF ratio, indicative of increased sFlt-1 or decreased PlGF levels, has been associated with adverse maternal and fetal outcomes in preeclampsia (Verlohren et al. 2010). While the lack of a significant difference in our study's comparison may seem surprising, it highlights the complexity of angiogenic factor dynamics in preeclampsia. Factors such as gestational age, disease severity, and individual variations can influence sFlt-1 and PlGF levels, contributing to variability in their ratios. This finding suggests a potential imbalance or dysregulation in the sFlt-1/PlGF ratio, which is often considered a critical factor in the pathophysiology of preeclampsia (Oladipo and Jayade 2024; Yagel et al. 2023). The sFlt-1/PlGF ratio is known to be altered in preeclampsia, with increased sFlt-1 and decreased PlGF contributing to the anti-angiogenic state associated with preeclampsia (De Oliveira et al. 2013; Gannoun et al. 2023). However, the lack of a statistically significant difference in this specific comparison in the preeclamptic group may imply variability within this cohort or a potential overlap in the ranges of sFlt-1 and PlGF values in this context.

We also report the following clinical demographics to be significantly different among the groups, maternal age (*p* < 0.0001), maternal weight (*p* < 0.0221), systolic (*p* < 0.0001), and diastolic (*p* < 0.0001) pressures, gestational weight (*p* < 0.0001) and gestational age (*p* < 0.0001). These findings suggest that these factors may have an impact on the development and severity of preeclampsia. Interestingly, there were no significant differences in terms of gravidity and parity between the study groups suggesting that the number of pregnancies or births may not be directly associated with the development of preeclampsia.

## Conclusion

In conclusion, this study provides compelling evidence of the differential expression of angiogenic factors sFlt-1 and PlGF in the placental bed of pregnancies complicated by preeclampsia (PE) and HIV infection in the era of ART. We observed a significant reduction in sFlt-1 and PlGF immunostaining in PE compared to normotensive pregnancies, highlighting the role of defective placentation in PE pathophysiology. Notably, sFlt-1 levels were significantly elevated in EOPE compared to LOPE and normotensive groups, implicating defective placentation in the dysregulation of angiogenic factors. Additionally, the impact of HIV on angiogenic balance was evident, with HIV-positive women showing higher sFlt-1 levels and altered PlGF expression, suggesting a complex interaction between HIV and PE in exacerbating angiogenic dysregulation. Despite the lack of significant differences in the sFlt-1/PlGF ratio, our findings reinforce the critical role of these biomarkers in understanding PE. This study is the first to examine the immunostaining of sFlt-1 and PlGF in the placental bed of HIV-positive PE patients of African ancestry, contributing valuable insights into the intricate mechanisms underlying PE in this population.

## Data Availability

No datasets were generated or analyzed during the current study.
